# Hydroxychloroquine in COVID-19: Potential Mechanism of Action Against SARS-CoV-2

**DOI:** 10.1007/s40495-020-00231-8

**Published:** 2020-08-24

**Authors:** Sairaj Satarker, Tejas Ahuja, Madhuparna Banerjee, Vignesh Balaji E, Shagun Dogra, Tushar Agarwal, Madhavan Nampoothiri

**Affiliations:** Department of Pharmacology, Manipal College of Pharmaceutical Sciences, Manipal Academy of Higher Education, Manipal, 576104 India

**Keywords:** COVID-19, Hydroxychloroquine, SARS-CoV-2, Coronavirus, Pandemic, QT prolongation

## Abstract

**Purpose of Review:**

The rapid spread of virus, severe acute respiratory syndrome coronavirus 2 (SARS-CoV-2), has turned out to be a global emergency. Symptoms of this viral infection, coronavirus disease 2019 (COVID-19), include mild infections of the upper respiratory tract, viral pneumonia, respiratory failure, multiple organ failure and death. Till date, no drugs have been discovered to treat COVID-19 patients, and therefore, a considerable amount of interest has been shown in repurposing the existing drugs.

**Recent Findings:**

Out of these drugs, chloroquine (CQ) and hydroxychloroquine (HCQ) have demonstrated positive results indicating a potential antiviral role against SARS-CoV-2. Its mechanism of action (MOA) includes the interference in the endocytic pathway, blockade of sialic acid receptors, restriction of pH mediated spike (S) protein cleavage at the angiotensin-converting enzyme 2 (ACE2) binding site and prevention of cytokine storm. Unfortunately, its adverse effects like gastrointestinal complications, retinopathy and QT interval prolongation are evident in treated COVID-19 patients. Yet, multiple clinical trials have been employed in several countries to evaluate its ability in turning into a needed drug in this pandemic.

**Summary:**

This review attempts to summarize the MOA of CQ/HCQ and its side effects. The existing literature hints that till date, the role of CQ/HCQ in COVID-19 may be sceptical, and further studies are warranted for obtaining a therapeutic option that could be effectively used across the world to rise out from this pandemic.

## Introduction

What was then an unknown cause of respiratory ailments in the patients of Wuhan, China, the disease has now grown into a lethal threat to mankind in the form of a pandemic. The World Health Organization (WHO) had declared it as a public health emergency of international concern on 31 January 2020 [1]. Initially referred to as the 2019 novel coronavirus (2019 n-CoV) [2–4] and controversially the Wuhan virus [5–7], it was later officially named by WHO as the severe acute respiratory syndrome coronavirus 2 (SARS-CoV-2) that is responsible for causing coronavirus disease 2019 (COVID-19) on 11 February 2020 [8].

The SARS-CoV-2 belongs to *Betacoronavirus* genera of the Coronaviridae family, which, along with the *Alphacoronavirus*, are known to infect mammals. In contrast, the remaining two members of the family, *Gammacoronavirus* and the *Deltacoronavirus*, infect avians [9]. They contain a positive strand of RNA with 29,903 nucleotides and 10 open reading frames where structural proteins like Spike (S) proteins, Nucleocapsid (N) proteins, Envelope (E) proteins and Membrane (M) proteins are coded by 3′ end, and polyproteins, namely, pp1a and pp1b, are coded by 5′ end [10]. The pp1a amd pp1b are cleaved into16 non-structural proteins (nsps) by two proteases namely papain like proteases and 3C like proteases [11]. The outer end of the virus structure inherits protruding spikes thus appearing like a crown-shaped virion [12••]. These spike glycoproteins are essential for the propagation of viruses into the human body through interactions with angiotensin-converting enzyme 2 (ACE2) to mediate viral pathogenicity [13].

SARS-CoV-2 is known to have an incubation period of about 2–14 days in humans [14]. It could transmit to individuals via respiratory droplets formed when symptomatic patients cough and sneeze [15]. Depending on the severity of infection, the infected individuals present symptoms including systemic disorders like dry cough, tiredness, headache, dyspnoea, lymphopenia and respiratory ailments like bilateral pneumonia, ground-glass opacity (GGO), sneezing and rhinorrhoea [16]. Certain patients are reported to have diarrhoea that could hint the effect of SARS-CoV-2 in the gastrointestinal tract and also increased transaminases and prothrombin times and reduced protein levels in blood indicating hepatic injury [17]. Severe cases in SARS-CoV-2 affected geriatric population showed acute respiratory distress syndrome (ARDS) that leads to septic shock. Computed tomography scans (CT scans) showed larger and denser lesions with prominent damage to the lungs [18]. Respiratory failure, cardiogenic shock and other multiple organ failures have been attributed to the deaths of infected patients [19]. Interestingly, it was seen that females were less susceptible to COVID-19 due to oestrogen that played an essential role in immunity [20]. The WHO has reported more than 2.5 lakh deaths and more than 3.5 million confirmed cases across the globe till date, and the development of effective therapeutics are need of the hour [21].

Currently, there are no specific treatments available for COVID-19 [22•]. With the clocks ticking and the fast-spreading nature of COVID-19, looking out for therapies through the repurposing of available drugs could be a quicker alternative. The antimalarial drugs chloroquine (CQ) and hydroxychloroquine (HCQ) have shown promising potential in clinical setups to combat COVID-19. Looking at the possibility of CQ/HCQ, in silico studies have been employed with molecules having scaffold similar to CQ [23] and repurposing of CQ for evaluating its effectiveness [24•]. HCQ is preferred due to its higher water solubility, lower toxicity and also feasibility for prolonged use with increased tolerance [25, 26]. HCQ is speculated to provide better treatment outcomes than CQ [27]. These drugs have shown interference in the endocytic pathway, blockade of sialic acid receptors, restriction of pH mediated S protein cleavage at the ACE2 binding site and prevention of cytokine storm. With these actions, they also have shown adverse events like gastrointestinal problems, retinopathy and QT prolongation. Having said this, the quick search for the COVID-19 therapy has pushed CQ/HCQ into the rapid deployment of its clinical trials to evaluate the safety and effectiveness. Given the fact that morbidity and mortality due to COVID-19 increase exponentially, there is a need to explore the evidence from the clinical trials. Thus, the present review describes the MOA, side effects and various clinical trials undergoing across the world using HCQ to target COVID-19.

### Inhibitory Effects of CQ and HCQ on the Potential Sites of SARS-CoV-2 Entry and Pathophysiology in Host Cells

CQ obtained from the bark of Cinchona trees has been widely used for a long time as an antimalarial agent [28]. In recent times, its hydroxyl derivative, HCQ, has proven to be safer than CQ due to the decreased renal and ocular toxicity and is being used as a substitute for CQ [29]. CQ/HCQ has shown its potential to destroy SARS-CoV-2 in the following ways.

#### Inhibition Through Interference in the Endocytic Pathway

**Fig. 1:**
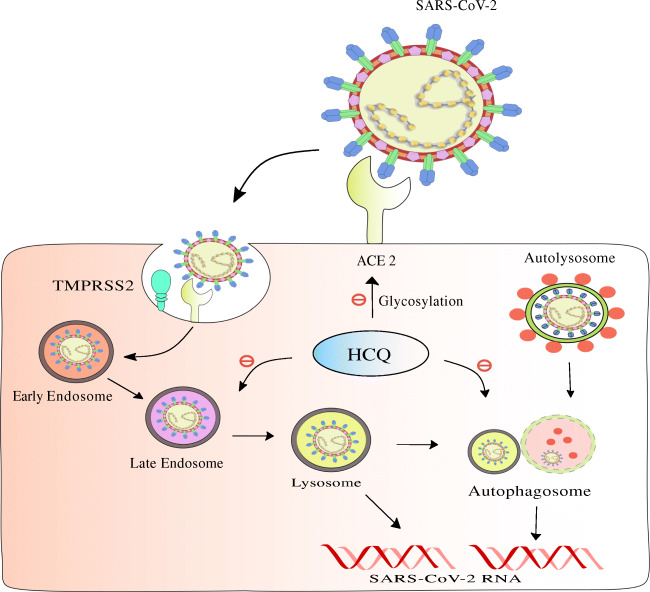
The role of CQ/HCQ in inhibition of ACE2 glycosylation, conversion of early endosome into late endosome and formation of autophagosome

SARS-CoV-2 facilitates their entry into the host cells through different ways via endocytic pathways. The nasal epithelium expresses dynamin, a GTPase that is essential for endocytosis mediated through clathrin-layered vesicles. Pneumocytes express CtBP1&2 and Pak1 proteins which are essential in micropinocytosis that forms an essential mediator in the pneumocyte endocytic pathway [30••]. CQ gathers in the endosomes and lysosomes and leads to pH neutralization that leads to hindrance on the effects of proteases, thus preventing S protein cleavage and, ultimately, the process of viral access into a host organism [31]. CQ inhibits fusion of lysosomes with autophagosomes due to dysregulation of syntaxin 17 (STX17) as shown in Fig. 1. Further, it hampers Golgi functioning and blocks transportation of material into lysosomes [32••]. HCQ also prevents the movement of SARS-CoV-2 from early endosomes to early lysosomes that are important for viral genome release [33••] as shown in Fig. 1. The rise in pH of lysosomes and endosomes mediated by HCQ further leads to formation of autophagosomes that break the S protein preventing the membrane fusion [34].

#### Inhibition Through Blockade of Sialic Acid Receptors

Recently, it has been identified that the N terminal of S protein in SARS-CoV-2 is similar to the region where sialic acid receptor binding occurs in MERS-CoV [35]. Therefore, SARS-CoV-2 may mediate its entry via these sialic acid receptors in the upper respiratory pathway and the previously known ACE2 receptor. Another study has also identified a novel binding site for the binding of gangliosides in the SARS-CoV-2 S protein N-terminal domain (NTD). Both CQ/HCQ were efficient in inhibiting the sialic acids, especialy the 9-O-SIA variant, however HCQ showed a better potency [36••]. The SARS-CoV-2 is also speculated to enter through binding with α 2–6-linkage and α 2–3-linkage sialic acid receptors. The 2–6-linkage is highly expressed in the epithelia of the conjunctiva and cornea, whereas the nasolacrimal region possesses both these receptors [37]. Therefore, the incident entry of viral particles into the eye or the respiratory passage via duct of nasolacrimal system may lead to its successful entry into the host cells.

#### Inhibition Through Restriction of pH Mediated S Protein Cleavage at the ACE2 Binding Site

The S proteins mediate the association and entry of viral particles in host cells [38]. It has two components, namely, the S1 subunit and S2 subunit [39•]. The S1 subunit is responsible for mediating entry into the host cells through attachment at the ACE2 receptor. Following this, the cellular proteases like transmembrane serine protease II (TMPRSS2) facilitate the S priming, which leads to cleavage of S1/S2 and S2’. Interestingly, it was reported that splice alterations occurring in the genome of TMPRSS2 due to single-nucleotide polymorphism could alter its expression levels, thus affecting rate of SARS-CoV-2 infection in patients [40]. Upon cleavage of the S2 domain, it releases fusion peptide [41]. This allows the viral membrane to fuse with the cellular membrane promoted by S2 subunit [42••]. A recent in silico study reported that SARS-CoV-2 spikes bound with a higher affinity to a truncated ACE2 as compared with full-length ACE2. This truncated ACE2 possessed the same conserved N-terminal region as that of full-length ACE2 [43]. Another study reported the critical amino acid residues that are responsible for enhanced affinity of spike binding to ACE2 [44•]. The ACE2 undergoes glycosylation for it to convert to an active form. Therefore, when SARS-CoV-2 S protein binds to it, the ACE2 receptor undergoes glycosylation and gets activated. In this case, CQ/HCQ plays an essential role where it prevents the glycosylation of ACE2 receptors, thus preventing entry of SARS-CoV-2 into the host organisms [45••] as shown in Fig. 1.

#### Inhibition Through Prevention of Cytokine Storm

**Fig. 2:**
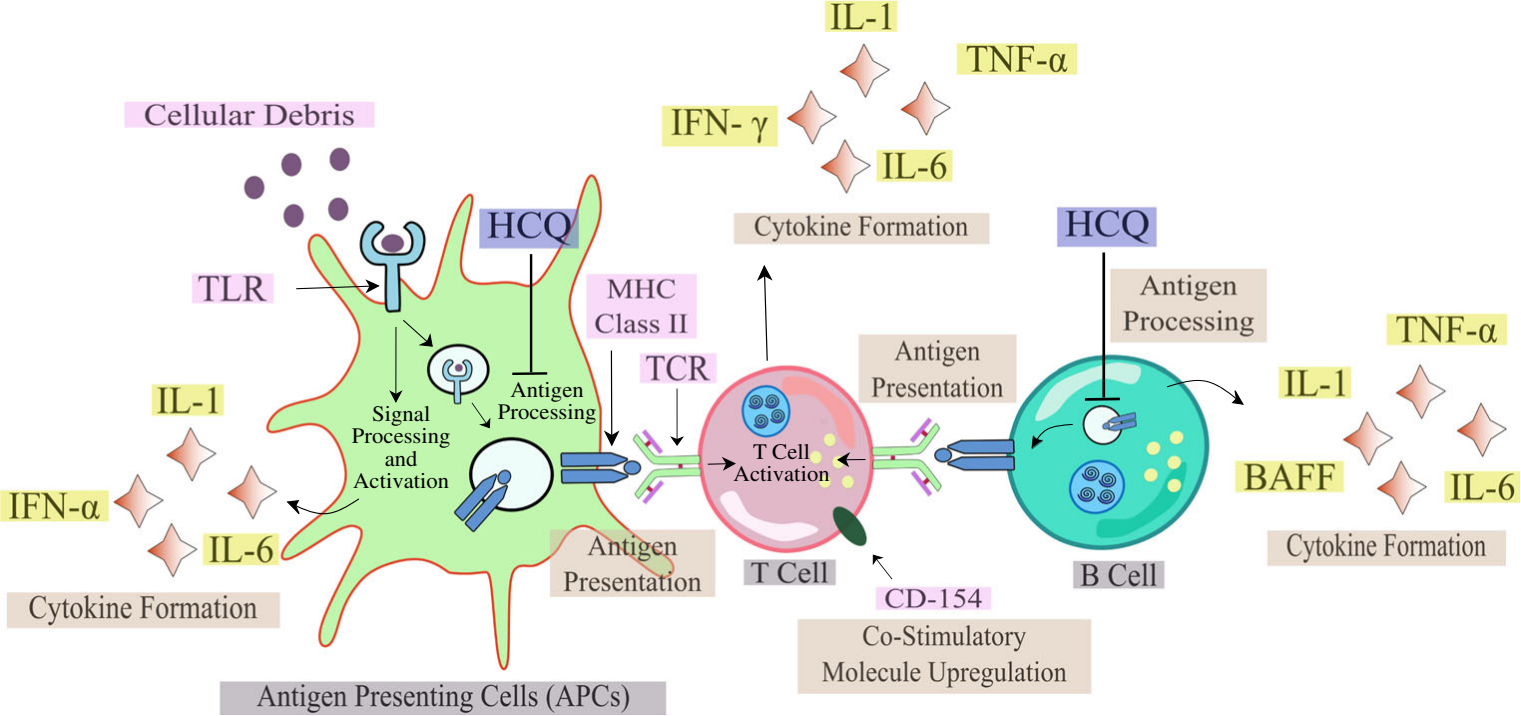
The role of HCQ in prevention of cytokine storm via inhibition of antigen processing and TLR receptor binding in antigen-presenting cells (APCs)

**Fig. 3:**
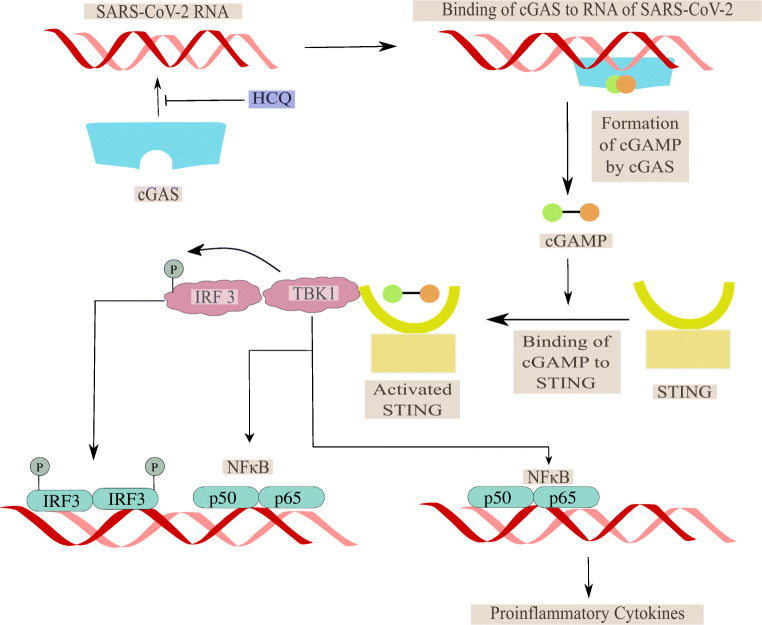
The role of HCQ in prevention of cytokine storm via inhibition of cGAS mediated release of proinflammatory cytokines

Recently, a striking feature was observed in severely ill patients in China whose plasma profiles showed elevated levels of cytokines [2]. Therefore, it would not be wrong to associate such an increase in levels of cytokines, termed as a cytokine storm, to grade how severe the disease is progressing. In the antigen-presenting cells (APC), HCQ inhibits the antigen processing and presentation of autoantigen mediated by the major histocompatibility complex (MHC) class II to T cells. Due to this, the levels of activated T cells decline, causing a reduction in the production of cytokines generated by T cells and the B cells [46, 47]. The changes in pH caused by HCQ also affects the toll-like receptor (TLR) functioning. Synergistically, HCQ can also associate with nucleic acids to block TLR9 binding and RNA facilitated activation of TLR 7 processing, therefore, reducing the production of cytokines [48], as shown in Fig. 2. The cyclic GMP-AMP (cGAMP) synthase (cGAS) upon binding to DNA leads to the formation of cGAMP. This cGAMP upon associating with stimulator of interferon gene protein (STING) leads to the generation of interferon 1 (IFN I) via the interferon regulatory transcription factor (IRF). HCQ inhibits the DNA binding at cGAS, thus preventing cytokine production and ultimately controlling cytokine storm [49] as shown in Fig. 3.

### Side Effects of CQ/HCQ

**Fig. 4:**
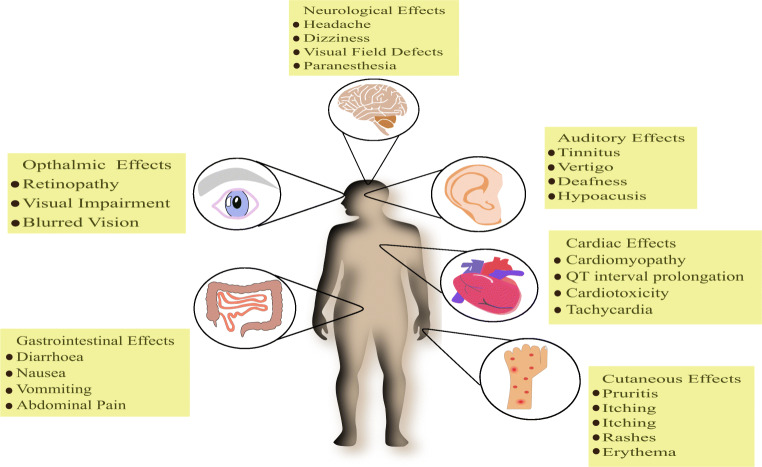
Summary of side effects exhibited by CQ/HCQ

The use of CQ/HCQ in COVID-19 patients has been owed to its immunomodulatory and antiviral properties but unfortunately still lacks the proof of their effectiveness. The use of HCQ does come with a set of adverse effects that cannot be waved off. The most commonly seen side effects of CQ/HCQ are gastrointestinal (GI) upset along with nausea and vomiting [50, 51]. GI symptoms basically include dyspepsia, dysgeusia, abdominal cramps, etc. Along with these, cases of rashes, itching, headaches and even tinnitus have been surfaced [52]. The severe side effects of CQ/HCQ include retinopathy [53, 54] and QT interval prolongation [55–57]. In the epithelia of the retinal pigment, the lysosomal deterioration of outer components of the photoreceptor by CQ/HCQ leads to retinal toxicity [58]. Ocular pigmentation could be possibly due to binding of these drugs to melanin that could damage ciliary bodies along with the cornea and the retina as well [59]. The use of CQ/HCQ has also been linked to pigmentation in patients, especially skin, ear and nose cartilage, trachea and joint tissue [60]. Conditions like eruptive toxic epidermal necrolysis, acute generalized exanthematous pustulosis, erythema multiforme and toxic epidermal necrolysis are seen in CQ/HCQ patients [61]. Cutaneous eruptions observed could be due to an imbalance in the immune system in CQ/HCQ-treated patients [62]. A summary of CQ/HCQ side effects is shown in Fig. 4.

### Clinical trials using HCQ as a treatment for COVID-19

Currently, a total of about 242 studies have been registered under clinicaltrial.gov that involve HCQ either as a single regimen or a combination regimen with other drugs. Approximately about 70 studies have not yet started recruiting subjects. A total of 18 studies are fully active but not yet recruiting, while 107 studies are recruiting the required subjects. Out of the 107 studies, 96 studies are of interventional design, while 11 are observational in nature. Some studies employ only HCQ (NCT04340544, NCT04351620, NCT04466540, NCT04435808), while some compare the efficacy of HCQ with other interventions like azithromycin (NCT04334382), ivermectin (NCT04391127), tocilizumab (NCT04332094) and favipiravir (NCT04411433). Further, a combination of drugs with HCQ is also included like HCQ + favipiravir (NCT04392973) and HCQ + azithromycin (NCT04336332). A total of 19 studies have been completed out of which one involves the use of convalescent plasma (NCT04441424). These studies have been mainly focused on the safety and efficacy of HCQ (NCT04321278), (NCT04261517) and (NCT04358068). It is worth noting that 14 studies have either been terminated or suspended. Few studies like NCT04323631, NCT04371926 and NCT04347512 were withdrawn mainly due to a rise in concerns of HCQ adverse events and on the advice of health authorities.

### Conflicting Reports of the Use of CQ/HCQ in Management of COVID-19

HCQ is looked upon as the wonder drug or miracle drug that showed antiviral property to aid in management of COVID-19 patients. CQ reduces fever and lung lesions and prolongs the progression of the disease, while HCQ in combination with azithromycin can reduce viral load in COVID-19 patients [63••]. Azithromycin enhances the capacity of HCQ in lowering viral load [63••]. CQ also reduces pneumonia exacerbations and duration of symptoms [64]. Even though the MOA of CQ and HCQ is the same, HCQ is more effective over CQ [65]. Nevertheless, contradictory data has also been published on HCQ that discourages its use as a potential therapeutic candidate in COVID-19 primarily owed to its multiple side effects as mentioned before in this article. HCQ also failed to show a beneficial result in an observational study [66]. Another report also claims that HCQ was not able to manage even mild cases of COVID-19 [67].

### NIH and WHO Verdict on the Usage of HCQ for treating COVID-19

In India, the Indian Council of Medical Research (ICMR) has approved the use of HCQ for prophylaxis of COVID-19 in asymptomatic healthcare workers and asymptomatic household contacts of confirmed cases [68]. Later, ICMR issued another advisory that permitted use of HCQ even for asymptomatic frontline non-healthcare workers [69], even though the US FDA has cautioned against the use of HCQ in COVID-19 patients owing to its side effects [70].

Similarly, the National Institute of Health (NIH) recently put a check on a study involving the efficacy of HCQ in COVID-19 patients with a note that even though it does not cause any harm, it did not provide additional benefits either [71]. These views bring a lot of conflicts that bring us to a position where we are still unable to conclude whether HCQ could be the game changer for COVID-19. As every coin has two sides, it becomes essential for us to understand both aspects of this drug. NIH conducted a study through the National Heart, Lung and Blood Institute to ascertain the effect of HCQ as compared with control hypothesizing beneficial role of HCQ (NCT04332991) in improving clinical conditions of patients in 15 days. It was a multicentre, blinded, placebo-controlled, randomized clinical trial. It had about 479 participants with a parallel assignment of two interventions, namely, HCQ 400 mg twice daily on day 1 followed by HCQ 200 mg on days 2–5 oral and placebo twice daily for 5 days. Primarily death, hospitalization on invasive mechanical ventilation and non-invasive ventilation were assessed. Ultimately due to the absence of any benefits for COVID-19 patients, NIH decided to stop the study.

Similarly, the WHO had set up a solidarity trial for finding an effective therapy for COVID-19 using HCQ and lopinavir/ritonavir drug interventions. It was concluded that very less or almost no reduction in death rates of COVID-19 patients were seen due to which it was stopped. Interestingly, it was also concluded that other studies involving HCQ could be continued, while this check was only applicable to the solidarity trial using HCQ [72].

## Conclusion

The COVID-19 pandemic has quickly grown to become one of the biggest threats that mankind has faced since the Influenza outbreak in the last century and is now being regarded as a ‘global healthcare emergency’. As the pandemic has grown stronger, it has called for quick and effective therapies where-in the role of efficient clinical trials is crucial for the search of the ‘game changer’ in therapeutics of COVID-19. As we have seen how CQ/HCQ can interfere in endocytic pathway, prevent ACE2 glycosylation, promote blockade of sialic acid receptors and reduce cytokine storm, this could have been attributed to its promising preliminary results in managing the viral symptoms. But it is seen that even though it is weighed down by its adverse effects like gastrointestinal indications, QT prolongation and retinopathy, it has been extensively employed in clinical trials across the globe. Results from a lot of recently initiated studies in some countries have shown positive outcome measures; however, the data is still not conclusive enough to establish this drug as the first-line therapy for treating/preventing COVID-19 infection, and more definitive results are still awaited.
